# An Inquiry into the Nature and Treatment of Gravel, Calculus, and Other Diseases Connected with a Deranged Operation of the Urinary Organs

**Published:** 1821-05

**Authors:** 


### CRITICAL ANALYSES

OF

### RECENT PUBLICATIONS, IN THE DIFFERENT BRANCHES OF MEDICINE AND SURGERY.

"I would have men know, that, though I reprehend the easie passing over of the causes of things "by ascribing them to secret and hidden vertues and properties; (for this hath arrested and laid "asleepe all true enquiry and indications;) yet I doe not understand but that, in the practical "part of knowledge, much will be left to experience and probation, whereunto indication cannot "so fully reach: and this not only in *specie*, but in *individuo*. Yet it was well said, *Vere scire esse per causas scire*."—BACON.

*An Inquiry into the Nature and Treatment of Gravel, Calculus, and other Diseases connected with a deranged Operation of the Urinary Organs.* By WILLIAM PROUT, M.D. F.R.S. 8vo. pp. 227.—London: Baldwin, Cradock, and Joy. 1821.

THE author of the present volume, it is said in the Preface, has been in the habit, for many years, of closely attending to the diseases of the urine; and this work exhibits an outline of his observations on the subject. "With his own observations," the author adds, "the principal facts and opinions of others have been likewise incorporated; while, on the other hand, to avoid controversy, whatever appeared doubtful has been in general omitted without remark. It was his original intention to prefix an historical introduction respecting the urine, with a detailed account of the chemical experiments on which many of his peculiar views are founded; but, upon reflection, he was induced to relinquish both these objects for the present, and to confine his attention chiefly to practical points. Chemical details could not, indeed, be altogether avoided, because chemistry constitutes the very basis on which the whole superstructure is founded: care, however, has been taken to render them as plain and concise as possible, and thus to present such a view of this part of the inquiry as may be intelligible to the general reader. To *establish* new views on medical subjects, is almost too much for an individual to hope. The author, therefore, has chiefly confined himself to *illustration;* and, leaving it to the profession at large to establish his conclusions, (if they are capable of being established,) rests in the mean time perfectly satisfied that justice will be done to his attempts."

The task of the reviewer on this occasion is a very simple and easy one: he has but little to do with criticism; he will best perform his duty by giving as perspicuous an account of the author's *illustrations*, or observations of facts, as his abilities will permit; but, as the space consigned to him will not allow of a comprehensive exposition of those observations, he must endeavour to present such a view of them as will show the principal points of the evidence on which the author's inferences are established: so that, when these are brought forward as the most probable inferences,—as they appear to be,—the reader of this abstract may recognize them to be such. The facts related are to a considerable extent original: their great importance will be best proved by showing how fertile they have already been rendered, by the reflections of the author, in apparently useful pathological inferences and indications for the practice of medicine.

The work commences with an "Introduction;" in which the author presents a contrasted view of the elementary principles of the human urine and blood. After giving a description of the properties of these fluids, the author cites Berzelius's account of the relative proportion of the several *elements* (as far as properly regards the fluid in question,) in the urine; which is as follows:

| | | |
|---|---|---|
| | Water | 933·00 |
| Animal and destructible principles. | Urea | 30·10 |
| | Lithic acid | 1·00 |
| | Pure lactic acid, lactate of ammonia, and animal matters not separable from these | 17·14 |
| | Mucus of the bladder | ·32 |
| Alkaline and earthy salts. | Sulphate of potash | 3·71 |
| | ——— of soda | 3·16 |
| | Phosphate of soda | 2·94 |
| | ——— of ammonia | 1·65 |
| | Muriate of soda | 4·45 |
| | ——— of ammonia | 1·50 |
| | Earthy phosphate, with a trace of fluate of lime | 1·00 |
| | Silex | ·03 |
| | | 1000·00 |

Healthy urine, when first voided, reddens litmus paper; and has, therefore, been generally considered as containing a free acid. Its specific gravity has been estimated, at a mean, to be about 1·0125.

Besides the principles just designated as the constituents of healthy urine, this fluid contains others, some of which, as well as some of those of healthy urine, do not manifest themselves in the blood. These relations are shown at one view in the following tabular arrangement of the constituents of the several fluids.

| Blood contains | Urine contains, *Healthy,* | Urine contains, *Diseased,* |
|---|---|---|
| Water | Water | ——— |
| Albumen, fibrin, red particles | ———<br>——— | Albumen. Fibrin. Red particles. |
| ——— | Urea | ——— |
| ——— | Lithic acid | ——— |
| ——— | ——— | Nitric acid. Erythric acid. Purpuric acid. Oxalic acid. Benzoic acid. Carbonic acid. Also xanthic oxide. Cystic oxide. Sugar. Bile. |
| ——— | ——— | |
| ——— | ——— | |
| ——— | ——— | |
| ——— | ——— | |
| ——— | ——— | |
| ——— | ——— | |
| ——— | ——— | |
| Lactic acid, and its accompanying animal matters | Lactic acid, and its accompanying animal matters | ———<br>———<br>——— |
| ——— | Mucus of the bladder | ——— |
| ——— | ——— | Pus. |
| Sulphur. Phosphorus. Muriatic acid. Fluorine? | Sulphuric acid<br>Phosphoric acid<br>Muriatic acid | ———<br>———<br>——— |
| ——— | Fluoric acid? | ——— |
| Potash. Soda. Lime. Magnesia. Silex? | Potash. Soda. Ammonia. Lime. Magnesia. Silex? | ———<br>——— |
| ——— | | ——— |

Having adduced those preliminary general observations, the author considers, in a particular manner, the properties of the several constituents of the urine, and the various conditions under which they present themselves. The *water* may exist in an increased or a lessened quantity in regard to the whole or part of the rest of the constituents, or its quantity may vary from the ordinary state with a correspondent variation of that of the whole or part of the other elements. Urine containing an increased proportion of water, is usually limpid and colourless, and may be easily known by its low specific gravity.

Albumen, fibrin, and the red particles, as already remarked, only appear in certain diseases. The albuminous matter is usually more analogous to that of the chyle than that of the blood: this curious fact will be noticed more particularly hereafter. "Urine containing chylous albumen is generally palecoloured, and, on being exposed to a temperature of about 150°, becomes opaque, and deposits this principle in a coagulated state. The effect is increased by the addition of an acid, especially the nitric acid; but the most delicate test of albumen is dilute acetic acid, and the prussiate of potash. *Bloody* urine is, of course, likewise albuminous; but is always more or less of a dark colour."

*Urea* is formed in the kidney, of some of the constituents of the blood, Dr. Prout says, "perhaps the albumen." The colour, and other sensible qualities of the urine, were formerly ascribed to this principle; but Berzelius proved that it is colourless, and Dr. Prout has shown* that it has no remarkable smell or taste. It commonly assumes the form of a four-sided prism. Its crystals are pellucid and colourless, and have a slight pearly lustre. The specific gravity of its crystals is about 1·350. Water at 60° dissolves more than its own weight of urea.

"An excess of urea in the urine seems to be characteristic of a peculiar form of disease, to be described hereafter. The mode which I commonly use to detect an excess, is to put a little of the urine into a watch-glass, and add to it carefully nearly an equal quantity of pure nitric acid, in such a manner that the acid shall subside to the lower part of the glass, from its greater specific gravity, and allow the urine to float above it. If spontaneous crystallization takes place, an excess of urea is indicated; and the difference of excess can be inferred, near enough for practical purposes, by the greater or less time which elapses before the crystallization takes place, which time may vary from a few minutes to two or three hours. Such urine is commonly, but not always, of a pale colour."

Dr. Prout says, he knows of no disease characterized by a diminished proportion of urea. In diabetes very little is present, but the other saline principles are also relatively deficient.

*Lithic*, or *uric*, acid, though not found in the blood, appears to be always present in healthy urine; and, in a pathological point of view, it seems to be the most important of its principles. Dr. Prout has some peculiar opinions respecting the mode of its existence in healthy urine, and the modifications which it is capable of undergoing, the arguments for which he exposes in detail. Berzelius appears to consider that it exists, at least in part, in the urine in a free state, and, consequently, that it is held in solution merely in virtue of its solubility in water; and this seems to be the general opinion. But, from its existing in the urine in a greater proportion than that in which it is soluble in water in a free state,—1000 parts of urine containing one part of lithic acid, according to Berzelius; and

* *Medico-Chirurgical Transactions*, viii. p. 529.

one part of this acid requiring at least 1720 parts of water at 60° (at which temperature uric acid is not precipitated from healthy urine,) to dissolve it, according to Dr. Henry, and at least 10,000 times, according to Dr. Prout,—and from several other reasons, some of which are the results of his proper experiments, Dr. Prout infers, as a probability, that the lithic acid in healthy urine exists in a state of combination with ammonia, (which combination is much more soluble than free lithic acid,) and that in reality urine contains no uncombined acid at all.

Lithic acid combined with diluted nitric acid forms what Brugnatelli described as a peculiar acid, under the term *erythric acid:* this compound may be obtained in the form of pellucid colourless crystals. The addition of ammonia to a solution of these crystals, produces a fine purple colour; the same solution treated with potash and sulphuric acid, in a way formerly described by Dr. Prout, (*Philos. Transac.* 1818,) produces *purpuric acid*, which assumes the form of a yellowish or cream-coloured powder. Such changes in the urine sometimes happen, in certain diseases, "either by the action of the kidney, or the natural operation of the various principles existing in the urine upon one another;" the mode of which operation Dr. Prout proceeds to attempt to explain. The amorphous, or uncrystalized, sediments, usually denominated *pink* and *lateritious* sediments, and supposed by Mr. Proust to constitute a peculiar acid, which he named *rosacic*, have been found by Dr. Prout to consist essentially of the *lithate of ammonia*, and sometimes of the lithate of *soda;* and he considers that they owe their colour partly to the colouring matter of the urine, (to be described hereafter,) and partly (in some instances almost entirely,) to the *purpurates of the same bases.* The reasons on which those inferences are founded are given in detail, and they are such as renders the truth of the author's inferences in the highest degree probable; but it cannot be absolutely proved, because the proportion in which the alkaline purpurates exist in the amorphous sediments—admitting their existence—is too small to permit them to be separated so that their presence may be demonstrated.

In some forms of disease, Dr. Prout says, the urine does really contain an uncombined acid, (which will be pointed out hereafter;) and in this case the lithate of ammonia is decomposed, and the lithic acid deposited in a crystalline form, and nearly pure; thus constituting the disease called *gravel.* When the compounds of the lithic acid exist abundantly in the urine, this fluid is almost invariably of a deep colour; and, if at the same time a free acid be present, the urine is for the most part unusually pellucid, and free from mucus.

*Oxalic acid* is present in some states of disease; but whether it is secreted in a free state is not known, "as it always occurs in combination with lime, which, from its great affinity for that earth, it may be supposed to get from the urine." The oxalate of lime, in some rare instances, forms gravel, and not unfrequently calculi.

*Benzoic acid* has been stated to exist, occasionally, in the urine of children; but this is doubted by Berzelius, and Dr. Prout has never met with an instance of its occurrence. There is, we believe, not the least reason for doubting the presence of this acid in the urine of some animals, as the horse and the cow.

*Carbonic acid*, it seems probable, from some experiments of Dr. MARCET, exists in the urine under certain circumstances; and Dr. Prout has seen small calculi composed principally of carbonate of lime.

*Xanthic oxide*, is the name given by Dr. Marcet to a substance constituting the chief bulk of a small renal calculus, and which appears to have been witnessed only in this one instance. It may be distinguished by the lemon *yellow* colour which it yields when treated with nitric acid; from which property it derives its name.

*Cystic oxide* has, in some rare cases, formed the entire substance of calculi: it has not been observed in any other state. It may be distinguished by its solubility in acids and alkalies, and by a peculiar odour which it yields when burned.

*Sugar* found in the urine of persons having diabetes, is said by Dr. Prout to differ in its appearance from common sugar, and approach more nearly to that of grapes. "This principle may be suspected to exist in *pale* urine, when its specific gravity is above 1·030."

*Bile* is sometimes present in the urine, in small proportions. "Such urine is generally of a deep brownish-red colour, when in considerable quantity, and viewed by transmitted light; but, when examined in small quantity, it has sometimes a greenish appearance." The most delicate and unequivocal test of its presence, according to Dr. Prout, is the addition of muriatic acid to the urine, which is by this means rendered green.

*Lactic acid*, and its accompanying animal matters, are considered by Berzelius to be the principles to which urine owes its proper colour and odour: it is to the presence of free lactic acid, principally, that the same chemist ascribes its property of reddening litmus paper. Dr. Prout says, "I would not be understood to deny either of these points; but I confess I have never been able to satisfy myself of them so completely as I could wish." The lithate of ammonia, when in solution, will redden litmus paper; and this combination is capable, Dr.

Prout observes, of existing in the same solution with a solution of super-phosphate of ammonia, which has likewise the power of reddening litmus paper. With respect to the colour of the urine, Dr. Prout says, it "appears to be sometimes owing, in part at least, to the presence of the purpurates. I admit, however, the existence of a colouring principle in the urine, besides the purpurates; but, as far as I know, it has never been obtained in a separate state. The colour of the urine, however, is liable to be modified by the nature of the ingesta, and a variety of other circumstances." Dr. Prout adduces several reasons for inferring that the urine derives its colour from two distinct principles. "The cause of the peculiar *smell* of the urine," he adds, "has never been explained; but it is probably connected with some undefinable compound, into which, if I am not mistaken, sulphur, phosphorus, and azote, largely enter."

*Mucus* is derived from the membrane lining the urinary passages. When it exists in an abundant quantity, and is inordinately adhesive, it becomes a matter of great importance, especially when the urine deposits the phosphates; as, by acting as a sort of cement, it renders them more liable to adhere together and form concretions.

*Pus* is almost always accompanied with an inordinate quantity of mucus, from which it can be distinguished, Dr. Prout acknowledges, not without great difficulty. The symptoms of the disease will commonly furnish the best means for determining the nature of a discharge of a purulent appearance.

*Sulphur. Sulphuric acid. Sulphates.*—Sulphur appears to be present in some peculiar state of combination in the urine; but by far the greatest proportion of this principle exists in the urine as sulphuric acid, in combination, of course, with alkaline substances. "I do not find," Dr. Prout says, "that sulphuric acid has ever been suspected of being concerned in the production of any morbid condition of the urine; and I believe it never has been observed to form (in combination with lime, for instance,) any sensible property of urinary calculi or gravel." —"I think, however," he adds, "that I have seen a case where the sulphuric acid, in a free state, acted as a precipitant of the lithic acid."

*Phosphorus. Phosphoric acid. Phosphates.*—Phosphorus, like sulphur, appears to exist in minute quantity in the urine as well as in the blood, and probably, like that substance also, as an element of some of the constituent principles of these fluids. Phosphoric acid is found in healthy urine in about the same proportion as sulphuric acid. We transcribe the rest of the author's remarks on this substance in detail.

"In a pathological point of view, phosphorus and *its* compounds particularly claim our attention. I am not acquainted with any disease connected with the simple absence of phosphorus and its compounds from the urine; though the existence of such a disease is not improbable, when we consider that health is always accompanied by the due separation of a certain proportion of these principles from the economy. On the contrary, cases where this acid exists in the urine in a free state, and apparently acts as a precipitant of the lithic acid, are by no means uncommon. Phosphoric acid, however, becomes most formidable when the earthy bases, lime and magnesia, are secreted in greater abundance than natural; which, by combining with the acid, form insoluble phosphates, and thus constitute by far the most distressing species of gravel and calculus. In healthy urine, this acid, like the sulphuric, appears to exist principally in union with potash, soda, and ammonia, and partly, perhaps, with lime and magnesia; the different salts being, from the excess of acid, in the state of superphosphates. Phosphoric acid is shown to exist in the urine, by its yielding, with the nitrate of barytes, a precipitate soluble in nitric acid, and again precipitable from that acid, by ammonia, without decomposition."

*Muriatic acid. Muriates.*—Muriatic acid exists in the urine, as well as in the blood, in combination with soda and potash: "thus appearing," Dr. Prout says, "to pass through the kidneys unchanged. This acid and its compounds, in a pathological point of view, are, perhaps, to be considered as the least important existing in the urine; no disease arising from their excess or defect being at present known."

*Fluoric acid* has been said by Berzelius to exist in the urine, in small quantity, combined with lime: but, Dr. Prout remarks that, as far as he knows, this observation has not been verified by any other chemist.

*Soda. Potash. Ammonia.*—The two fixed alkalies exist in the urine, as well as in the blood, in combination with the sulphuric, phosphoric, muriatic, and, according to Berzelius, the lactic, acids. "Ammonia exists only in the urine, apparently in combination with the muriatic, phosphoric, and lithic acids. No disease is known to arise from the excess or defect of the fixed alkalies; but the deposition of the earthy phosphates in the urine is almost always accompanied, if not immediately produced, by an excess of ammonia. Hence, in a pathological point of view, this is a principle of the greatest importance. The apparent source of the excess of ammonia is the urea."

*Lime. Magnesia. Silex.*—The author's observations respecting these substances cannot be given otherwise than in detail.

"Lime and magnesia exist both in the blood and the urine; but in very different states. In the blood, they appear to enter, perhaps as elements, into the composition of the albuminous principles; and hence cannot be obtained without combustion: in the urine, they occur chiefly in the saline state, apparently, as before observed, in union with the phosphoric acid. I am not acquainted with any disease characterized by a deficiency of these earths in the urine; but the most distressing and dangerous form of calculous complaints is connected with, and indeed immediately arises from, their *excess*,—namely, the deposition of the earthy phosphates. In this form of the disease, the earthy bases seem to be separated in a much greater proportion than usual; while the quantity of phosporic acid is relatively diminished. This deficiency of phosphoric acid, however, does not seem to arise from a deficiency of phosphorus, but from some defect in the oxygenating operation of the kidneys, by which that principle is permitted to pass through them unchanged: for the urine, under these circumstances, often seems to contain, in some unknown state of combination, even more phosphorus than natural. The urea, also, in this form of disease, exists in great abundance; but in some peculiar and apparently imperfect state, by which it is rendered extremely prone to decomposition, and liable to be converted into the carbonate of ammonia. Hence, the urine in this disease is either naturally alkaline, or speedily becomes so; and this excess of alkali contributes to the rapid union of the earthy bases with the phosphoric acid present, and their consequent deposition in the form of phosphates. Urine containing an excess of the phosphates is generally of a pale colour."

"Silex," Dr. Prout says, "has been stated to constitute urinary sediments, and even to form a part of urinary calculi in some instances: but this assertion requires to be better authenticated than it is at present, before it can deserve credit. This earth, however, ordinarily exists in the urine in minute quantity, according to Berzelius; but he supposes it to be derived from the water which we drink, which is not improbable."

As this part of the work comprises so many principles which are constantly referred to in the subsequent divisions of it, we have thought it necessary to give a comprehensive abstract,—the means for testing the presence of the principles of the urine being alone wholly unnoticed, in the greater number of instances, because they are, in these instances, simply conformable with the general principles of chemical analysis;—and we shall, for the same reason, transcribe the whole of the author's reflections on the facts which have been passed in review.

"From the preceding sketch, we find that the most striking differences between the blood and the urine, is the complicated nature of the latter. The astonishing variety of substances formed from such a paucity of materials, naturally leads us to reflect upon the vast extent of the operation of the kidneys. On considering, however, a little more attentively the nature of the operations of these organs, we shall find, as Berzelius has justly remarked, that *acidification* constitutes the chief feature in them. Thus, the sulphur and phosphorus of the blood are converted by the kidneys into sulphuric and phosphoric acids: a new acid, the lithic, is generated altogether, &c. Such, then, evidently is the natural and healthy operation of these glands. We find, however, that, in certain forms of disease, this acidifying tendency is carried to excess, and nitric acid, oxalic acid, &c. are produced. On the other hand, it is occasionally suspended, diminished, or altogether subverted; and unchanged blood, or albuminous matter,—neutral substances, as urea or sugar,—or even alkaline substances, as ammonia, lime, and magnesia, are separated in abundance,—and the phosphorus and sulphur at the same time pass through the kidneys without being acidified. With respect to the character of the diseases attending these states of the urine, it will be generally found that, when acids are generated in excess, the urine is commonly small in quantity and high coloured, and the disease inflammatory: when neutral or alkaline substances, the urine, on the contrary, is generally pale coloured and larger in quantity, and the diseases are those of irritation and debility.

"With respect to the mode in which all the different substances existing in the urine are naturally combined, it is impossible to state any thing with certainty, except generally that the several acids divide the alkaline bases among themselves in the order of their respective affinities and quantities. The greatest difficulty which occurs among the salts, is with respect to the phosphoric and lithic acids and their compounds. There can be no doubt, however, as formerly stated, that the whole or both of these acids are in combination with some base or bases; otherwise the lithic acid could not be retained in solution. Yet the solution of these compounds reddens litmus paper very strongly; showing that the acids, though in a state of *combination*, are not in a state of *neutralization*, (two very different things, though frequently confounded with each other;) and we can only explain this by supposing that the affinity of the elements of the different salts are so balanced, that the ammonia of the super-lithate of ammonia, for example, is held too firmly in combination by its acid to be separated by the phosphoric acid of the super-phosphates."

The "*diseases of the urine*," the author remarks, are so extensively related with morbid conditions of the animal economy that there is some difficulty, at least in the present state of our knowledge, in devising an unexceptionable mode of arranging them. Perhaps, he adds, the most simple and obvious principle of arrangement is that founded on the *solubility* or *insolubility* of the elements met with in the urine. This he adopts, and, in conformity with it, he divides the subject into two sections:

"I. Diseases in which principles soluble in the urine are morbidly deranged in quantity or quality.

"II. Diseases in which principles insoluble in that secretion are similarly deranged.

"By this arrangement, we shall, indeed, as will be found hereafter, separate one or two diseases which appear to be closely connected with one another; but this defect, the consequences of which can be easily obviated, appears to me more than counterbalanced by the general conveniency of the arrangement in other respects.

"Under the first of the above general divisions will be considered,

"1. Various forms of albuminous urine.

"2. Anonymous diseases, in which an excess of urea is the characteristic symptom.

"3. Diabetes.

"Under the second,—all the various forms of gravel and calculus."

Dr. Prout does not, however, consider that the above list comprises all the morbid conditions of the urine; but they are all those of which his knowledge is sufficiently precise to be laid before the public. An important circumstance in the history of morbid states of the urine, is a diminution or an increase of it, or more expressly, a diminished or increased separation of *water* by the kidney, without regard to the quantity of the other constituents. These diversities of proportion are connected with two very different states of the system. "A diminished flow of urine," the author observes, "accompanies active inflammation, and an inflammatory state of the system in general. The urine is invariably of a deep colour. An increased flow of urine, or *diuresis*, very constantly accompanies those diseases connected with a peculiar state of nervous irritability, as hysteria. It may be also produced by certain passions of the mind, as fear. Lastly, it may be induced by local irritants acting on the urinary organs themselves. In those cases the urine is always of a pale colour."

Under the title of *diseases in which the presence of an albuminous principle is the characteristic symptom*, the author does not comprise urine rendered albuminous by the presence of blood, but "a peculiar condition of that secretion, in which it is found to contain one or more principles, usually more resembling those met with in the chyle than in the blood." These principles sometimes exist in very large proportions, in which case the urine undergoes a kind of spontaneous coagulation; but it is more frequently smaller, when the urine is almost invariably pale coloured, and of a moderate or low specific gravity. Dr. Prout thinks that mucus derived from the prostate gland, which may be coagulated by heat, has sometimes been mistaken for albumen. Mucus is coagulated by dilute acetic acid, which is not the case with albumen.

"The symptoms usually attending this albuminous condition of the urine, are those of irritability. In slighter cases, there is generally a frequent desire to pass water, and for the most part decided diuresis. I have never known albuminous urine attended by positive pain; though the patient, for the most part, complains of certain indescribable sensations, which render him conscious that all is not right. In severe cases, where the drainage from the system is greater than natural, there are, as might be expected, an inordinate craving for food, and other symptoms somewhat resembling diabetes."

The history of a case, presenting several very interesting circumstances, in which this state of the urine existed in an extreme degree, is then given in detail. When this case occurred to the knowledge of the author, he had already formed the notion that *chyle*, and not blood, is occasionally the source of the albuminous principle; he was therefore well prepared to recognize all the phenomena which seem to point out such an origin. Another case is then given, in which the albumen was less abundant, and which he observed before the notion just mentioned occurred to him; the history of which, nevertheless, presents similar indications. The author concludes his observations and reasonings on this point with remarking that, "however well-founded or important the fact of the presence of chyle in the urine may be, I cannot venture at present to draw any conclusion from it."

We may be permitted to request the reader to bear this circumstance in mind during the perusal of the review of the work of Dr. ALARD, in a subsequent part of this Journal, and to connect it with the fact that the colour, odour, &c. of certain substances taken into the stomach are found in the urine and not in the blood,—with the apparently well-authenticated cases of vomiting of urine,—and with some observations respecting the udder of the cow; which will appear in the next number of this Journal. Here will, we think, be presented what may warrant some new researches for communications between different parts, by means of absorbent vessels, that probably exist, and have not yet been discovered.

The slighter degrees of albuminous urine," Dr. Prout remarks, "in which the kidney may be considered as simply *passive*," may exist for years, without apparently producing any serious effects in the constitution.

Dr. Blackall attempted to show that, when the urine is albuminous in dropsy, the use of blood-letting in general is indicated; and Dr. Wells had already remarked that albuminous urine is "connected with too great action in some part of the system." Dr. Prout does not deny that blood-letting may here be proper; but, unless there are other symptoms present which seem to warrant that measure, he thinks that it should not be resorted to; and he seems to consider that, speaking generally, opium will prove more beneficial in this disease. He believes that the presence of albumen in the urine is, in some instances, the first step towards the conditions in which there is *excess of urea* and *diabetes*.

Whenever the specific gravity of the urine is high,—for example, above 1·025 or 1·030,—the proportion of urea, in common with the other principles, is necessarily larger than natural; and in this case crystallization will frequently take place, on the addition of nitric acid. "This concentrated state of the urine not unfrequently takes place in febrile and other diseases, and is quite unconnected with any disease of the urinary organs, and appears to depend upon a diminished secretion of water only:" and, perhaps, we may add, from the water of the urine being partly removed after it has arrived in the bladder. In other cases, " an excess of urea, as compared with the other ingredients of the urine, is actually present."

" Those diseases in which an excess of urea may be considered as in some degree characteristic, do not appear to have been hitherto distinguished, but have been probably confounded with other diseases, and particularly with that form of diabetes which has been sometimes denominated diabetes *insipidus.* These diseases, however, differ considerably from diabetes, as the following observations will show.

" The average specific gravity of the urine in these complaints seems to be a little above 1·020, and occasionally to vary from 1·015 to nearly 1·030. Most generally it is pale, but occasionally it is high coloured, and exhibits somewhat the appearance of porter, more or less diluted with water; and this variety in appearance not unfrequently takes place in the urine of the same person. When first voided, it reddens litmus paper. For the most part, it is entirely free from sediment, except the mucous cloud of healthy urine; and the only remarkable property which it appears to possess, is that of containing abundance of urea; so that, on the addition of nitric acid, crystallization speedily takes place. From the quantity of urea present, it is very prone to decomposition, and soon becomes alkaline, especially in warm weather."

This state of the urine appears, as far as is at present known, to be referrible to no peculiar symptoms, either constitutional or local, excepting, of course, in regard to the latter, the frequent evacuation of the urine; which is, in some instances, effected more frequently than is explicable by the distension of the bladder. " In the few cases of this disease which have hitherto fallen under my own immediate observation," says Dr. Prout, " the subjects have been middle-aged men, of thin and spare habit, with a sort of hollow-eyed anxiety of expression in their countenance; free from gout and constitutional diseases in general; and, as far as could be ascertained, free from any organic defect in the urinary organs." The author has had no opportunities of ascertaining the progress of these diseases; but he thinks it extremely probable that, if permitted to proceed, some of them will terminate in diabetes, or in a deposition of the earthy phosphates. Their causes being so little understood, and their nature and origin being probably various, the proper mode of treatment for them cannot be determined, and must be founded on remote and more or less vague analogies. " In every case, however," Dr. Prout says, " which has hitherto fallen under my own observation or knowledge, sedatives, and particularly opium, have been the only efficient remedies." Two selected cases are related, which show that much alleviation, at least, of the disease may be obtained by such medicines, combined with others appropriate for the removal of particular symptoms which may be present. Dr. Prout was induced to have recourse to opium in this complaint, from knowing the good effects of this medicine in diabetes; with which, as already remarked, he considers it to bear a close analogy.

The term *diabetes* is applied by Dr. Prout, after some other authors, exclusively "to those affections in which the urine is *saccharine.*" The urine is here almost always of a pale straw-colour; its specific gravity has been stated to vary from 1·020 to 1·050. Dr. Prout says he has seen it higher than this, but never so low. We select the following from amongst the author's proper observations on this subject, as the most interesting of them.

"The quantity of urea is almost always very much diminished, though I have never met with a specimen in which it was entirely absent. It contains, for the most part, little or no lithic acid. The usual saline matters existing in healthy urine, are met with in diabetic urine in nearly the same relative proportions, but their absolute quantity is very much diminished. Sometimes diabetic urine contains a little blood;* and not unfrequently albuminous matter analogous to that of the chyle. I have seen it also contain a white milky-like fluid precisely similar to chyle, which slowly subsided to the bottom of the vessel. In this case the vinous fermentative process was indeed very rapidly in the urine, the chylous matter apparently acting like yeast."

It has not yet been proved that a saccharine condition of the urine may exist without an increase of the *quantity* of this fluid; but Dr. Prout thinks it probable that it does exist, in its incipient stages, for example, when the symptoms may be overlooked. He thinks it doubtful whether the increased quantity of the urine is a consequence of the simple saccharine condition, or whether it depends on other causes; but he remarks, in a note, that "the most probable cause of the increased flow of urine, is that irritable state of the system which forms a part of the disease, and which appears to resemble nearly that peculiar condition sometimes present in hysteria and other nervous affections, in which a large flow of limpid urine frequently takes place."

Two cases in which amaurosis accompanied diabetes from the first, and which apparently originated from the same conditions of the system as the latter affection, were related in a late Number of this Journal. We have seen a case where it was apparently related, in a similar manner, with a considerable degree of deafness.

Whether or not the quantity of the urine is the consequence

* See WATT's Cases of Diabetes, pp. 47, 74.

of its saccharine condition, it seems, Dr. Prout says, to be in some degree a measure of the severity of the disease; for, the greater the flow of urine, the greater, for the most part, is its specific gravity and the proportion of sugar which it contains. Dr. Prout has suggested nothing original respecting the etiology of this affection. Nothing satisfactory has yet been advanced on this point. The cases adduced by Dr. Prout concur, with others which have been treated by the same medicine, to show the utility of opium, in large doses, which seems to be the most effectual measure hitherto employed. The advantages from animal diet and blood-letting, are not so well established: these means have lessened the quantity of the urine, but it is not certain that they have improved its quality. "I think, however," Dr. Prout remarks, "that there are stronger grounds for presuming that blood-letting has improved the quality of the urine, than that animal diet has produced this change."

The author commences his considerations on "*the diseases of the urine in which principles insoluble in that secretion are morbidly deranged in quantity or quality*," with a description of urinary gravel and calculi, and a summary account of their chemical composition, &c.

Substances deposited from the urine, though composed of the same general ingredients, may, the author says, in a pathological point of view, be conveniently divided into three classes: "1. Pulverulent, or amorphous sediments; 2. Crystalline sediments, usually denominated gravel; and, 3. Solid concretions, or calculi formed by the aggregation of these sediments." Amorphous sediments almost universally exist in a state of solution in the urine before it is discharged, and even afterwards until it begins to cool. Their colour is, for the most part, red, diluted with more or less of brown or yellow: they contain, at different times, almost every principle capable of becoming solid itself, or of forming a solid compound with any other principle of the urine.

"Generally speaking, however, they may be stated to consist of two species of neutral saline compounds,—viz. the lithates of ammonia, soda, and lime, tinged more or less with the colouring principle of the urine, and with the purpurates of the same bases, and constituting what are usually denominated *pink* and *lateritious* sediments; and, secondly, the earthy phosphates, namely, the phosphate of lime and the triple phosphate of magnesia and ammonia, constituting for the most parts sediments nearly white. These two species of sediments very frequently occur mixed together, though the lithates generally prevail: and it is to this circumstance, and to the little tendency that the salts of which they are composed have to assume the crystalline form, that their heterogeneous and amorphous nature is to be referred."

Crystalline sediments are composed of—1. Lithic acid nearly pure, (always more or less of a red colour;) 2. The triple phosphate of magnesia and ammonia, (always white;) and, 3. Oxalate of lime, (of a dark blackish-green colour.) These different varieties of crystalline deposits are never voided together, though they not unfrequently occur with amorphous sediments.

With respect to the appearances and chemical properties of urinary calculi, the author adds nothing of much importance to what had been said by Dr. Marcet.

The author next treats of the comparative prevalency of the different forms of urinary deposits, and the order of their succession, accompanying his relation of facts with remarks illustrative of the etiology of those affections. He, in the first place, adduces particular accounts of the results of examinations of the largest collections of calculi in England, which he afterwards exhibits in a general view in the following table. The names connected with the specimens of calculi examined, are those of the chemists who examined them.

| General character. | Particular Species. | Hunterian Museum, Mr. Brande. | Norwich, Dr. Marcet. | Guy's Hospital, Dr. Marcet. | Manchester, Dr. Henry. | Bristol, Mr. Smith. | Particular Totals. | General Totals. |
|---|---|---|---|---|---|---|---|---|
| *Lithic acid* | Nearly pure | 16 | 66 | 16 | | | 98 | |
| | Mixed with a little oxalate of lime | .... | .... | 6 | 71 | 74 | 6 | 294 |
| | Mixed with a little of the phosphates | 45 | .... | .... | | | 45 | |
| *Mulberry* | or oxalate of lime | 6 | 41 | 22 | 11 | 33 | 113 | 113 |
| *Cystic oxide* | .... | .... | .... | 1 | 2 | .... | 3 | 3 |
| *Phosphates* | Nearly pure | 12 | .... | .... | 4 | .... | 16 | |
| | Mixed with a small proportion of the lithic acid | 66 | .... | .... | 18 | .... | 84 | |
| | Phosphate of lime, nearly pure | .... | 4 | 3 | .... | 1 | 8 | 202 |
| | Triple phosphate, nearly pure | .... | .... | 2 | .... | 1 | 3 | |
| | Fusible, or mixed calculi | .... | 49 | 24 | .... | 18 | 91 | |
| *Alternating Calculi* | Lithic and mulberry | .... | 15 | .... | .... | .... | 15 | |
| | Mulberry and lithic | .... | .... | .... | 11 | 29 | 40 | |
| | Lithic and phosphates | .... | .... | .... | 39 | 12 | 51 | |
| | Mulberry and phosphates | .... | 1 | .... | 16 | 32 | 49 | |
| | Lithic, mulberry, and phosphates | .... | .... | .... | .... | .... | .... | 136 |
| | Mulberry, lithic, and phosphates | 5 | .... | .... | 7 | .... | 12 | |
| | Fusible and lithic | .... | 1 | .... | .... | .... | 1 | |
| | Fusible and mulberry | .... | 2 | .... | .... | .... | 2 | |
| | Composition not mentioned | .... | .... | 6 | .... | 10 | 16 | |
| *Comp. Calculi* | Mixture not mentioned | .... | 2 | 7 | 3 | 8 | 25 | 25 |
| | | 150 | 181 | 87 | 187 | 218 | | 823 |

Our extracts from the author's remarks on the above data must be more partial than those we have given in respect to former subjects: those remarks will not admit of a perspicuous abstract; we shall, however, select a few of such as appear of most importance to medical practitioners in general.

It appears, from the foregoing table, that somewhat more than one-third of the whole number of calculi, in the collections referred to, belongs to the class in which lithic acid predominates: but, when it is considered that the whole of the calculi examined by Dr. Marcet were not sawed through,—that others were examined in a way not calculated to lead to precise results in this point of view,—and that it is universally admitted, by all authors on this subject, that lithic acid constitutes by far the most common nucleus round which other calculous matter is deposited,—Dr. Prout thinks that it may be safely asserted that "at least *two-thirds* of the whole number of calculi originate from lithic acid: that is to say, if a lithic acid nucleus had not been formed and detained in the bladder, two persons at least out of three who suffer from calculus would never have been troubled with that affection." Dr. Prout points out the diversity of the proportion of *mulberry* calculi in the different collections, as a curious subject that cannot at present be accounted for. The proportion of calculi consisting chiefly of phosphates, appears, from the table, to be about one-fourth of the whole; but, from the mode in which some of them were examined,—some not being sawed through, and Mr. Brande, after having cut through them, having removed a portion of the whole cut surface by a file, in which way "all the different ingredients of the calculi were obtained" for examination,—it cannot be known whether several of those consisting chiefly of the phosphates had not a lithic acid nucleus. In the Manchester collection, where this point was regarded in the examination, Dr. Henry says that, "in four instances only out of 187, the calculus has been composed throughout of the earthy phosphates."

The causes above noticed have also rendered the general results respecting the proportions of *alternating calculi* equivocal. The stated proportion of calculi of this species is hence found to vary more than what is probably really the case: thus, it is said to be only about one-fourteenth in the collection at Guy's Hospital, whilst it is one-half in that at the Bristol Infirmary. This, Dr. Prout shows, in another part of the work, to be a very interesting subject in a pathological point of view. He arranges the alternations of the layers of different calculous matters in the following manner:—1. Lithic and mulberry; 2. Mulberry and lithic; 3. Lithic and phosphates; 4. Mulberry and phosphates; 5. Lithic, mulberry, and phosphates; 6. Mulberry, lithic, and phosphates; 7. Fusible and lithic; 8. Fusible and mulberry. The specimens of *compound calculi*,—that is, those "composed of different ingredients mixed up together," are comparatively rare.

Notwithstanding the diversities in their composition and appearance, above enumerated, urinary calculi may, Dr. Prout remarks, "be considered as made up of four elementary substances only, viz.—1. The lithic acid and its compounds; 2. The oxalate of lime; 3. The cystic oxide; and, 4. The earthy phosphates; two or more of which principles are seldom or never found in excess in the urine at the same time. Hence they may be supposed to represent so many distinct diatheses, or conditions of the system requiring to be separately considered; and this accordingly is the principle on which the future arrangement of my subject will be founded."

Having explained more particularly his reasons for adopting this arrangement, the author proceeds to treat "of the lithic acid diathesis in general, and on the best means of counteracting it, so as to prevent the original formation of calculus, or its recurrence after an operation." The perusal of this disquisition must, very generally, be productive of extreme gratification, for many reasons, amongst which, not the least interesting one is this, that the subject is discussed by a thorough chemist, who has not permitted his knowledge of this science, and the peculiar zeal he entertains for it, to give an undue bias to his views in physiology.

When, from particular causes affecting the health, the quantity of lithate of ammonia in the urine is increased above the natural standard, the excess is precipitated as the urine cools, and thus constitutes one (the *amorphous*) of the two forms of depositions of compounds in which lithic acid predominates. When such a pulverulent sediment appears, then, the obvious inference is that there is an excess of lithic acid in the urine; and such is most generally the case, though not universally so, for the compounds of lithic acid appear to be sometimes deposited in consequence of a very slight excess of some other acid in the urine. The causes of this excess of lithic acid are stated by the author to be of three kinds:—"*a*. Simple errors in diet; *b*. Unusual or unnatural exercise, either bodily or mental, particularly after eating, and the want of proper exercise at all other times; and, *c*. Debilitating circumstances." We must refer the reader to the work itself for details on these points; we can only adduce some hints of the general inferences of the author respecting their mode of agency. He considers that they, in the first place, derange, in some unknown way, the digestive functions, by means of which either chyle too imperfect for the purposes of the economy, or some new and unnatural principle is generated, which, requiring to be removed from the system, is eliminated by the kidney, and by that organ converted into lithate of ammonia; and thus an *excess* of this substance is formed.

Several useful inferences and indications are derived by the author from the *colour* of the amorphous sediments, which he distinguishes into three principal varieties:—1. Yellowish or nut-brown; 2. Reddish-brown or lateritious; and, 3. Pink, sediments.* Those characterized by the nut-brown colour, (that of ripe hazle-nuts,) consist essentially of the lithate of ammonia, but usually contain also more or less of the phosphates; and generally, but not at all constantly, the nearer they approach to white, the greater is the proportion of the latter substances. "This class of sediments," the author remarks, "may be termed the *sediments of health*, if the term may be allowed,—being such as are produced in the urine of healthy or slightly-dyspeptic individuals by errors of diet, and all the other circumstances before mentioned, which seem, independently of actual fever, to produce turbid urine." When there is, however, an extraordinary liability to this affection, a tendency to excess of lithic acid is denoted that may lead to gravel and its consequences, of which it is almost constantly the forerunner.

The sediments of the second class vary in tint from nearly white (when they are with difficulty distinguished from the former,) to a deep brick-red or brown. They consist essentially of the lithate of ammonia, or lithate of soda, tinged with a large proportion of the colouring principles of the urine, and more or less of the purpurates of ammonia and soda. When the purpurates exist in the urine (indicating the secretion of nitric acid by the kidney,) "feverish or inflammatory action is almost constantly indicated;" and this law is so constant, the author adds, that he has never seen a decided exception to it. Amongst the diseases in which the colouring matter of the urine is secreted in a greater abundance than ordinary, the author mentions gout, rheumatism, and "hepatic affections."

The pink sediments consist essentially of the lithate of ammonia, but they differ from those of the two former classes in being almost entirely devoid of the yellow tint derived from the colouring matter of the urine; and, consequently, in owing their colour chiefly to the purpurate of ammonia. "This class of sediments, therefore," the author says, "appears to indicate the absence of the large proportion of the colouring principle

* A plate is attached to the work, in which the tints of the principal varieties of those several classes of sediments, as they generally appear, are represented.

of the urine, so constantly present in inflammatory fever; and to denote the secretion of a greater quantity of nitric acid, and the consequent formation of more of the purpurate of ammonia: and this view of the subject actually coincides with my observations. The most perfect specimens of this kind of sediment which I have ever seen, were obtained from the urine of dropsical individuals: they occur also occasionally in the urine of the hectic, and of those obviously labouring under certain chronic visceral affections, especially of the liver."

The presence of these sediments in the urine, the author considers, appears to show that fever has existed and is going off, rather than that it exists at present. They appear almost constantly in continued fevers; but this, he presumes, can be explained "upon the supposition that the sediments, for example, generated by the fever of yesterday, appear in the urine secreted during the remission of to-day; and those generated to-day, in the urine of to-morrow, &c." The length of time which the urine is retained by feverish patients must also be taken into consideration.

When discussing in a particular manner, in this part of the work, the causes of crystallized sediments or gravel, the author illustrates the operation of a free acid in the urine, by remarking that the addition of a few drops of any acid to healthy urine will produce a deposit of crystallized lithic acid: so that an excess of lithic acid is not always, though it is generally, present when this acid is precipitated from the urine. "I have frequently," says Dr. Prout, "seen the urine so completely divested of lithic acid in this form of disease, that, upon adding to it even an excess of a mineral acid, not another particle of lithic acid has been deposited." The free acid productive of this phenomenon is not always the same: "most generally," Dr. Prout says, "it appears to be the phosphoric acid, sometimes the sulphuric. I think I have also seen it take place from the nitric and erythric acids, and occasionally from some other acid of a destructible nature, which I was unable to make out. Even the carbonic acid may be occasionally the cause of this precipitation."

On treating of the conditions of the system under which gravel is formed, the author remarks that the symptoms in most instances are such as indicate some error in the digestive functions,—as acidity of the stomach, flatulence, &c.

Having discussed the history of this affection, Dr. Prout proceeds to consider the means by which urinary sediments may be obviated. In respect to the first class of amorphous sediments, he remarks that, when they occur only occasionally and not frequently, the affection "scarcely requires a formal treatment with medicine, but a careful attention on the part of the patient to avoid all the circumstances (errors of regimen and the like,) which have been before stated to frequently give origin to this deposite in the predisposed." The treatment of the second and third classes must be absolutely regulated by, and adapted to, the nature of the disease of which they are a consequence. The treatment of gravel must be chiefly directed by the same principles. The use of alkalies is, however, for the most part, particularly indicated; "but they are seldom or never to be given alone, but, to be really useful, must be conjoined with alteratives and purgatives. The pil. submur. hydrarg. comp. or a pill composed of the pil. hydrarg. and antimonial powder, taken at night, and followed up the next morning by a solution of Rochelle salts and carb. of soda in a bitter infusion, may be had recourse to. A little of the same mixture may be taken two or three times a-day, so as to keep the bowels fairly open; or, instead of this, a little magnesia may be taken in a glass of soda-water, as often as it may be found necessary."

In the treatment of the most severe stage of these affections, or what is usually denominated a *fit of the gravel*,—in which there is a union of the second class of amorphous with crystalline sediments, which, according to Dr. Prout, indicate fever and inflammation, as well as the secretion of a great excess of lithic acid,—the principles of the treatment are similar to those proper for the milder stages; only that blood-letting, general or local, or both; antimony, opium, or hyosciamus, in larger quantities than in the latter, are here also indicated; and Dr. Prout thinks it a matter of much importance that such measures should "precede the use of diuretic medicines."

With respect to the causes of the deposition of oxalate of lime, Dr. Prout remarks, we know but very little, the data are so imperfect: "I shall, however," he adds, "venture to make a few remarks upon the subject, which I leave my readers, if they choose, to consider as *conjectural.*

"1. We have seen that the oxalate of lime frequently forms renal calculi, which often increase to a considerable size in the bladder. From this I think we may infer, that the formation of this concretion is connected with a distinct diathesis excluding the existence of other diatheses; and that it is not an accidental occurrence happening in common with many others to the urine.

"2. From the dissection of calculi, it appears that the oxalate of lime diathesis is both preceded and followed by the lithic acid diathesis; a circumstance which is peculiar to these two forms of deposite, and which, when taken in conjunction with the phenomena and circumstances already related, appears to show that they are of the same general nature: consequently it is probable that,—

"3. In this diathesis, instead of lithic acid, the oxalic acid is generated; which, by combining with the lime naturally existing in the urine, forms the concretions in question.

"4. Is this oxalic acid actually secreted by the kidney, or is it formed afterwards by the action of the nitric acid upon some of the other constituents of the urine in the same manner that the purpurates appear to be formed? From various reasons which might be mentioned, the former opinion seems more probable.

"From all these observations taken together, I am induced to conclude that the oxalate of lime diathesis, though consisting in a manifestly deranged action of the kidney, and therefore distinct from the lithic acid diathesis, is nevertheless of the same general nature; and consequently that it requires a mode of treatment founded upon the same general principles."

After having given a summary of the cases of cystic-oxide calculi related by Dr. Marcet and Mr. Brande, the author adduces the following remarks:

"We know nothing of the state of the urine in this diathesis, or whether the calculous matter is ever deposited in the form of gravel or other sediment; but the probability seems against this supposition. We know very little, also, respecting the state of the general health; though I think we may conclude that it is not much affected, and that the cause of the disorder is rather a diseased or depraved action of the kidney.

"With respect to the medical treatment of this diathesis, we are equally ignorant. If the above view be adopted, that it is of the same general nature as the lithic acid diathesis, the nature of the mode of treatment will be obvious from what has already been said: but this must be decided by future observation."

The author's remarks on the "phosphatic diathesis," will not admit of a perspicuous abridgment: we shall extract from them some particulars which appear of very remarkable importance. After a general account of the constitutional symptoms, which "consist in great irritability of the system, and derangement of the chylopoietic viscera in general," and a description of the appearances of the urine, in cases where amorphous sediments of the phosphates are deposited as the urine cools and has stood for a little time, the author remarks that "in all cases the urine is extremely prone to decomposition, becomes alkaline by the evolution of ammonia, and emits a most disgusting smell." The greater proportion of the cases which have come under his own observation, have been "distinctly traced to *some injury of the back*, (as a fall from a horse or the like.)" It is an old observation, he remarks, that injuries of the back produce alkaline urine. Dr. Prout says he has not had an opportunity of inspecting a body after death under these circumstances. We have ourselves seen alkaline urine, from abundance of ammonia, in three cases where inflammation (shown by dissection,) of the spinal marrow occurred from other causes than mechanical violence. In one case,—the subject of which was a player on the clarionet, engaged at Vauxhall, and who had sweat much, from playing, during a cold and windy night, previously to the attack of the disease,—the sweat, which was copious, had also a very strong ammoniacal odour. This patient had paraplegia. The sweat and urine often smell strongly of ammonia in tetanus, which appears to be always accompanied with inflammation of the spinal marrow.

Dr. Prout is of opinion that excess and deposition of the phosphates will also take place from local causes, which may be "some irritation about the bladder or urethra, especially when operating constantly for a considerable length of time;" but, in the case of the origin of this species of sediment from foreign bodies being present in the bladder, round which they are collected, he does not admit the explanation usually given of this circumstance to be generally true;—that is to say, that the urine in contact with the foreign substances always *undergoes an incipient process of decomposition.* The fact is, he says, "that the foreign substance sympathetically affects the kidney, and produces a change in the urine, causing it to abound in the phosphates, which are deposited on the foreign substance." Dr. Prout does not notice the experiments of exposing foreign substances to urine without the bladder,—exposed to air and undergoing decomposition,—the results of which have been supposed to favour the prevalent opinion. We presume he has not thought such inapt experiments worthy of his attention. "With respect to the proximate cause of this form of disease," he says, "we may suppose it to consist in a diminished or suspended action of the usual acidifying powers of the kidneys, and the formation, instead of lithic acid, of a greater quantity of alkaline matter than natural,—as urea (equivalent to ammonia), lime, and magnesia: but this being little more than a simple expression of obvious facts, of course throws no light upon the immediate cause of these depraved actions." He alludes to the notion that the mucous membrane of the bladder is the source of the phosphatic sediments, and says he does not deny this altogether; and even thinks it possible that, in some instances, the earthy matter may be partly derived from this source.

The *crystallized* sediments composed of the phosphates, are constituted almost invariably of the triple phosphate of magnesia and ammonia, and exist in the form of perfectly-white shining crystals. This form may exist alone, or accompany the amorphous sediments of the phosphates: it is a disease of a milder character than the latter, and often precedes it. The urine is pale coloured, and, upon standing for some time, an iridescent pellicle is frequently formed upon its surface, which is composed chiefly of the salt in question. Minute crystals of the same salt are frequently attached to the sides of the vessel in which the urine has stood for some time. When the urine much abounds with it, the crystalline deposit is formed before the urine is discharged from the bladder.

The causes of this affection "resemble, or may perhaps be identical in all respects, with those occasioning the preceding affection;" they may, however, be much slighter in degree. Mental anxiety will frequently produce, in many people, an excess of this salt in the urine. Several medicines that act as diuretics will cause it; "as the neutral salts in some cases, and particularly the Rochelle salts, and other saline compounds in which the acid is of vegetable origin." The author thinks it likely that a long-continued use of alkaline remedies will produce a tendency to an excess of the phosphates in general. This affection is more frequent in children than in adults. "The indications of cure to be attended to in these forms of disease, appear to be two: to diminish the unnatural irritability of the system; and to restore the state of the general health, and particularly of the urinary organs, by tonics and other appropriate remedies." In severe cases, especially of the amorphous species of sediment, "opium," says the author, "as far as my experience has hitherto extended, is the only remedy that can be employed with any advantage to fulfil the first indication." It must be given in doses of from one to five grains three times a-day. In conjunction with the opium, when the more distressing symptoms have been alleviated, the mineral acids, or citric acid, cinchona, uva ursi, and iron, may be had recourse to. When distressing pain is felt in the loins, a large pitch or galbanum plaster may be applied. When purgatives are requisite, they should be of the milder kind, as small doses of castor-oil. The author is doubtful whether mercury should be at all employed in the more severe cases; if it is used, it should be merely as "an alterative." All diuretics, as well as alkaline remedies, should be avoided. "The diet, in severe cases, should be of the mildest and most nutritious kind, and taken in very moderate quantities at a time." The author thinks an animal diet preferable, in general, to one of acescent vegetables, commonly recommended. Every thing that can be done for the patient is "of very little use, if the mind cannot be set at rest."

This chapter terminates with the following observations respecting those intermediate or *transition* states which usually exist for some time during the change from other diatheses to the phosphatic, or which sometimes precede the phosphatic diathesis.

" *Transition from the lithic to the phosphatic diathesis.*—The first circumstances in the condition of the urine which generally denote a change from the lithic acid to the phosphatic diathesis, are the general paleness of its colour, and sometimes its increased quantity. There is also, for the most part, a great tendency in the urine, from the slightest causes, to deposit the amorphous sediments, which are always of a pale colour, and generally contain more or less of the phosphates intermixed with them. As the tendency to change proceeds, the urine may be frequently observed, after standing a few hours, to be covered with an iridescent pellicle on its surface, which, on examination, is found to consist principally of the triple phosphate of magnesia and ammonia; and, if at this time it be suffered to remain at rest for a short time, especially in warm weather, it becomes putrid, assumes a yellowish opaque appearance, and will be frequently found to contain large spicular crystals of the triple phosphate above mentioned. This constitutes what may be considered as the first stage of the series of changes in question. I have once or twice known a calculus extracted from the bladder during this stage, which I have had an opportunity of examining; and in every instance found it externally composed of pale-coloured lithate of ammonia nearly pure.

" The above state of the urine frequently occurs in sickly children, in whom the functions of the digestive organs are much deranged. It is liable also to occur from all the causes formerly enumerated, and particularly in those of an irritable habit, and who are subject to lithic deposites in general: also from any cause deranging the general health, or producing local irritation in the urinary organs. As to the constitutional affections, they are always more or less of the irritable kind, and generally accompanied by derangements of the digestive organs. In adults, also, there is not unfrequently some uneasiness felt in the region of the kidney. With respect to the tendency and danger of this sort of change, it may be generally removed, or at least prevented from getting worse, by a judicious use of the means formerly mentioned, provided its exciting causes can be removed. But, if these are permitted to operate, or are of such a nature that their operation cannot be prevented, medicines are of very little use; and the phosphatic diathesis will certainly sooner or later be induced, particularly if there be already calculus in the bladder.

In the second stage of the change in question, the urine commonly assumes a more decidedly pale whey-like colour, and is either alkaline when voided, or very soon becomes so. The lithate of ammonia also diminishes in quantity, or entirely disappears; while that of the phosphates, and particularly the triple phosphate of magnesia and ammonia, is increased. In short, this stage runs into the confirmed phosphatic diathesis by such imperceptible grades, that it is frequently difficult or unnecessary to draw the line of distinction; the symptoms and treatment being the same in most instances, only differing, perhaps, a little in degree. It may, however, be proper to observe, that where the lithate of ammonia is deposited in large quantity, mixed with the phosphates, hyosciamus rather than opium is to be preferred, as opium seems frequently to increase the formation of lithic acid.

"*Transition from the oxalate of lime to the phosphatic diathesis.*—In the second chapter of this section I have given a summary description of a calculus composed of a nucleus of oxalate of lime surrounded by the phosphates, with an account of the series of intermediate changes which took place. From this description it appears that the first step towards the change in question, was a secretion of an excess of lime; and that, as this proceeded, the proportion of oxalic acid decreased, while that of the phosphoric acid increased, until at length phosphate of lime, in nearly a pure state, was secreted, which constituted the external crust of the calculus. I had no opportunity of examining the urine in this case in the earlier stages of the affection; but in the latter stages it had all the properties, as might be expected, of that secretion when the phosphatic diathesis is present.

"With respect to the treatment, &c. to be adopted in this form of the disease, I have nothing to add to what has been already advanced.

"No instance in which a calculus of cystic oxide has been surrounded by the phosphates, has come to my knowledge."

The sixth chapter treats "on the modes of formation and future increase of calculi; on the symptoms produced by the different varieties in different situations; and on the medical treatment to be adopted when they are lodged in different situations."

On the first of these subjects there is but little that is peculiar to the author, that had not been indicated in the previous parts of the work, excepting some conjectures respecting the causes by which the bases of calculi are collected in a crystalline form in the kidney. The points of most practical importance in the other subjects of this chapter, were also pointed out in the discussions on the several calculous diatheses, or are familiar to medical practitioners.

With respect to the operation of *solvents* of calculi in the urinary passages, the author says he knows nothing; and, from what little he has seen, he is very much disposed to doubt if they can be ever so administered as to produce the desired effect.

The work concludes with "general observations on the periods of life, sex, climate, &c. most subject to calculous affections; on the mortality attending the operation of lithotomy; with observations on the circumstances in which it ought or ought not to be recommended:" the data for which, excepting those relating to the circumstances under which lithotomy is advisable, are collected from the writings of Dr. MARCET and Mr. SMITH.

